# Molecular analysis and immunological characterization of a founder mutation causing ARPC1B deficiency

**DOI:** 10.1038/s41435-025-00368-w

**Published:** 2025-11-17

**Authors:** Megan M. Dobrose, Meltem Ece Kars, Jareb J. Perez-Caraballo, Colleen M. Roark, Christine Mariskanish, Oscar Zavaleta-Martinez, Noemi Gomez-Hernandez, Saul Oswaldo Lugo-Reyes, Yuval Itan, Lizbeth Blancas-Galicia, Rubén Martínez-Barricarte

**Affiliations:** 1 Division of Genetic Medicine, Department of Medicine, Vanderbilt University Medical Center, Nashville, TN USA; 2 Division of Molecular Pathogenesis, Department of Pathology, Microbiology, and Immunology, Vanderbilt University Medical Center, Nashville, TN USA; 3 The Charles Bronfman Institute for Personalized Medicine, Icahn School of Medicine at Mount Sinai, New York, NY USA; 4 Allergy and Immunology Department, ISSEMYN, Toluca, Estado de México Mexico; 5 Allergy and Clinical Immunology Service, Unidad Médica de Alta Especialidad, Centro Médico Nacional de Occidente IMSS, Guadalajara, Jalisco Mexico; 6 Immunodeficiency Laboratory, National Institute of Pediatrics, Mexico City, Mexico; 7 Department of Genetics and Genomic Sciences, Icahn School of Medicine at Mount Sinai, New York, NY USA; 8 Mindich Child Health and Development Institute, Icahn School of Medicine at Mount Sinai, New York, NY USA; 9 Windreich Department of Artificial Intelligence and Human Health, Icahn School of Medicine at Mount Sinai, New York, NY USA; 10 Vanderbilt Center for Immunobiology, Vanderbilt University Medical Center, Nashville, TN USA; 11 Vanderbilt Genetics Institute, Vanderbilt University Medical Center, Nashville, TN USA; 12 Vanderbilt Institute for Infection, Immunology and Inflammation, Nashville, TN USA

**Keywords:** Disease genetics, Immunogenetics

## Abstract

Actin-Related Protein Complex 1B (ARPC1B) is a subunit of the ARP2/3 complex that is predominately expressed in hematopoietic cells and is involved in the regulation of actin polymerization. ARPC1B deficiency leads to combined immunodeficiency (CID) with symptoms of eczema, allergies, inflammation, recurrent infection, and thrombocytopenia. We characterize the disease-causing variant c.899_944del (p.E300Gfs*7) on the *ARPC1B* gene that originated from a founder effect in an indigenous American population. We showed that this variant impairs protein expression leading to a complete deficiency of ARPC1B. Additionally, we used mass cytometry to thoroughly analyze the effects of this mutation on the frequencies of immune populations. Our findings suggest that ARPC1B is critical for class switching since our ARPC1B-deficient patient had reduced frequencies of class-switched memory B cells. Furthermore, the frequencies of total CD4^+^, CD8^+^, and γδ T cells were reduced, consistent with an essential function of ARPC1B in T cell development. Overall, this study advances the knowledge of the c.899_944del *ARPC1B* mutation and the understanding of the role of ARPC1B in the immune system.

## Introduction

Combined immunodeficiency (CID) is a group of monogenic and phenotypically heterogeneous diseases that result in decreased presence or function of T lymphocyte immunity, sometimes combined with impaired presence or function of B lymphocytes or natural killer cells [1]. There are approximately 130 genes that are currently known to cause CID when mutated [2, 3]. In 2017, several cases of a new CID were reported, caused by mutations in the gene *Actin-Related Protein Complex 1B (ARPC1B)* that result in abolished protein expression [4, 5]. Since 2017, over 20 CID-causing variants in *ARPC1B* have been identified [6–9]. ARPC1B deficiency is characterized clinically by a range of symptoms including eczema, allergies, inflammation, recurrent infection, and thrombocytopenia [10]. ARPC1B is a subunit in the ARP2/3 complex, which is activated by the WASP protein and acts as a template for the formation of new actin filaments by branching from an existing filament to create y-shaped polymerization [11]. The ARP2/3 complex contains seven subunits including ARPC1, which exists as two isoforms in humans: ARPC1A and ARPC1B. The ARPC1B subunit is predominantly expressed in hematopoietic cells [5] and is composed of six WD40 domains that form a β-propeller [4]. ARPC1B is important for the regulation of actin polymerization, as it acts as a binding site for the VCA domain of WASP which initiates filament nucleation [12].

Over the last decade, next-generation sequencing has become increasingly more accessible, allowing for increased rates of diagnostic efficiency and changes in clinical treatment for patients with rare genetic diseases [13, 14]. Even with recent advances in next-generation sequencing, determining genetic variants to diagnose CID can be a challenging process. Frequently used genomic databases are predominantly based on European ancestry, so allele frequencies specific to other populations are not well catalogued [15, 16]. Therefore, patients of non-European descent are more likely to receive incorrect genetic diagnoses or have their genetic variants labeled as variants of uncertain significance (VUS) [17]. These genetic analysis challenges combined with the wide range of symptoms that patients with CID experience can lead to delayed diagnosis and treatment [18]. Furthermore, in diseases like ARPC1B deficiency in which only a limited number of patients with the disease are known, diagnosis can be even more difficult as the complete clinical presentation may not be fully described. These challenges enhance the need to better understand the genetics underlying this disease.

## Materials and methods

### Clinical diagnosis

The two patients’ clinical cases have previously been published [6, 9]. Briefly, Patient 1 first presented with symptoms as an infant, including abscesses, eczema, and keloid scars. Throughout his life, he suffered from recurrent pneumonia, fevers, IgG deficiency, and thrombocytopenia. In 2024, the patient died after experiencing a pulmonary hemorrhage following a severe episode of pneumonia.

Patient 2 suffered from severe eczema since infancy. Throughout childhood, he presented with multiple abscesses, including one on the face and neck that required surgical treatment. He developed a keloid scar at the incision site. He later underwent hematopoietic stem cell transplantation and is stable.

### Genetic analysis

Genomic DNA was extracted from the whole blood of the patients and their families using the GeneJET Whole Blood Genomic DNA Purification Kit (Thermo Fisher Scientific, Waltham, MA, USA). The mutation was identified by Invitae’s inborn errors of immunity gene panel. The region of interest on exon 8 of ARPC1B was amplified by PCR from genomic DNA from the patients, their families, and healthy individuals and Sanger sequenced to confirm the mutation. The primers designed to amplify this region are shown in Supplementary Table 1.

Whole genome sequencing was performed on genomic DNA from the patients and their families using Illumina short-read sequencing. Raw reads were aligned to human reference genome build hg38 using BWA-MEM v.0.7.17 [19]. PCR duplicates were identified in the aligned reads, base quality scores were recalibrated, and gVCF files were generated using Genome Analysis Toolkit (GATK, 4.2.5.0) by following the Best Practices Workflow [20]. gVCF files from ten individuals were jointly genotyped using GATK. Genotypes with an allele balance < 0.2 or > 0.8, a read depth < 10, or a genotype quality < 20 were set to missing using BCFtools [21]. (Variants with any missingness across ten individuals were excluded). KING was employed to infer within- and between-family relationships [22].

Distribution of Consensus Negative Selection (CoNeS) score for APRC1B was obtained using the method developed by Rapaport et al. [23] to quantify the strength of negative selection in genes throughout the genome. We used the published data in this paper to plot CoNeS values for genes known to cause IEI in an autosomal dominant, autosomal recessive, or both modes of inheritance to depict the score for ARPC1B relative to other genes.

### Ancestry inference

Genetic ancestry was determined using 1,500 samples with African, Amerindian, Indigenous American, and European ancestry from the Simons Genome Diversity Project (SGDP) [24] and 1000 Genomes Project (1KGP) [25] datasets as reference. Variants were filtered based on Hardy-Weinberg equilibrium (*P* < 1 × 10^−6^), and autosomal variants with a minor allele frequency > 1% were retained. Subsequently, variants were pruned for linkage disequilibrium (*r*^2^ = 0.2) using PLINK v2 [26]. PCA was performed on 324,939 pruned variants with smartpca implemented in EIGENSOFT version 8.0.0 [27]. Eigenvectors were calculated from 1,500 reference samples, and the study samples were projected onto these eigenvectors. The same steps were followed to conduct a second PCA using 20,251 variants from chromosome 7.

### Age estimation of *ARPC1B* c.899_944del

Chromosome 7 variants of the study samples were phased using SHAPEIT5 [28]with a reference panel of 329 Amerindian samples from 1KGP. First, common variants were phased based on pedigree information to generate a scaffold, onto which rare variants were subsequently phased. Phased variants were spot-checked for concordance with BAM files using IGV genome browser [29]. Haplotypes surrounding the variant site were determined based on pedigree information. The lengths of the haplotypes upstream and downstream of the mutation site on chromosome 7 were calculated in centimorgans (cM) and used as inputs for the Mutation_age_estimation.R script developed by Gandolfo et al. [30], which applies a maximum likelihood estimation method based on a Gamma distribution model to estimate the age of a mutation and construct confidence intervals. A confidence coefficient of 0.95 was applied with the option *chance sharing correction* = *FALSE*, assuming a correlated genealogy.

### ARPC1B overexpression

We generated expression plasmids containing C terminally His tagged versions of wild-type (WT) and mutant *ARPC1B*. Briefly, mRNA from Jurkat cells (ATCC, Manassas, VA, USA) was extracted using a RNeasy Plus Mini Kit (New England Biolabs, Ipswich, MA, USA) and retrotranscribed using a Verso cDNA Synthesis Kit (Thermo Fisher Scientific, Waltham, MA, USA). This cDNA was used to amplify WT *ARPC1B* while the mutant allele was synthetized by Genewiz Azenta Life Sciences (South Plainfield, NJ, USA). WT and mutant *ARPC1B* cDNAs were amplified with the primers shown in Supplementary Table 1, that introduced a His tag in the C terminal, using Q5 High-Fidelity DNA Polymerase (New England Biolabs, Ipswich, MA, USA). The PCR products were cloned into the pcDNA3.1 plasmid using the pcDNA™3.1-directional TOPO™ TA Cloning™ Kit (Invitrogen, Waltham, MA, USA), following manufacturer’s instructions. The sequences were confirmed by Sanger using primers in Supplementary Table 1.

Human Embryonic Kidney 293 T (HEK293T, ATCC, Manassas, VA, USA) cells were kept in culture in Dulbecco’s Modified Eagle Medium (DMEM) with 10% Fetal Bovine Serum (FBS). 500,000 cells were seeded in 6 well plates 24 h before transfection. Then, cells were either left non-transfected or were transfected using Lipofectamine 2000 (Thermo Fisher Scientific, Waltham, MA, USA) with 14 mg of each plasmid containing WT or mutant C-terminal His tagged *ARPC1B* or an empty vector as a control. Media was changed after 24 h. Cell lysates were collected after 48 h using RIPA buffer with a Pierce™ protease inhibitor tablet (Thermo Fisher Scientific, Waltham, MA, USA). Then, lysates were quantified using the Detergent Compatible Protein Assay (Bio-Rad, Hercules, CA, USA).

### Western blot

25 mg of each cell lysate was separated via sodium dodecyl sulfate-polyacrylamide gel electrophoresis (SDS-PAGE) using NuPAGE™ 4–12% Bis-Tris gels (Thermo Fisher Scientific, Waltham, MA, USA). They were then transferred onto polyvinylidene fluoride (PVDF) membranes (MilliporeSigma, Burlington, MA, USA). The membranes were probed with anti-His tag mouse antibody (J099B12, Biolegend, San Diego, CA, USA), anti-ARPC1B rabbit antibody (HPA004832, MilliporeSigma, Burlington, MA, USA), and after with α-tubulin mouse antibody (Vanderbilt Antibody and Protein Resource, Nashville, TN, USA). HRP conjugated anti-mouse secondary antibody (62-6520, Invitrogen, Waltham, MA, USA) and anti-rabbit secondary (AP156P, MilliporeSigma, Burlington, MA, USA) were used for protein detection. Membranes were developed with Pierce™ ECL Western Blotting Substrate (Thermo Fisher Scientific, Waltham, MA, USA) and imaged on the iBright™1500 imaging system (Invitrogen, Waltham, MA, USA). This experiment was repeated three times.

### RT-qPCR

RNA was extracted from the transfected HEK293T cells described above using the Qiagen RNeasy Plus Mini Kit (Qiagen, Hilden, Germany). RT-qPCR was performed using the Luna Universal One-Step RT-qPCR Kit (New England Biolabs, Ipswich, MA, USA) in a Bio-Rad CFX Real-Time PCR machine (Bio-Rad, Hercules, CA, USA). *ARPC1B* mRNA expression was calculated relative to *GUS* expression. Primers used to amplify *ARPC1B* and *GUS* are shown in Supplementary Table 1. This experiment was repeated three times with three technical replicates per condition each time.

### Cytometry by time of flight (CyTOF)

Peripheral Blood Mononuclear Cells (PBMCs) were extracted from the whole blood of P1, his family members, and eight healthy donors using Ficoll-Hypaque density gradient centrifugation (Amersham-Pharmacia-Biotech, Buckinghamshire, UK). P1 was 32 years old at the time of the blood draw, and the healthy donors were adults, age 25–40 years old. An equal number of female and male healthy controls were used for this experiment. Staining and data analysis were performed as described previously with the antibody panel shown in Supplementary Table 2 and the gating strategy shown in Supplementary Fig. 1 [31].

## Results

### The c.899_944del variant in *ARPC1B* originates from a founder effect of indigenous American ancestry

**Fig. 1:**
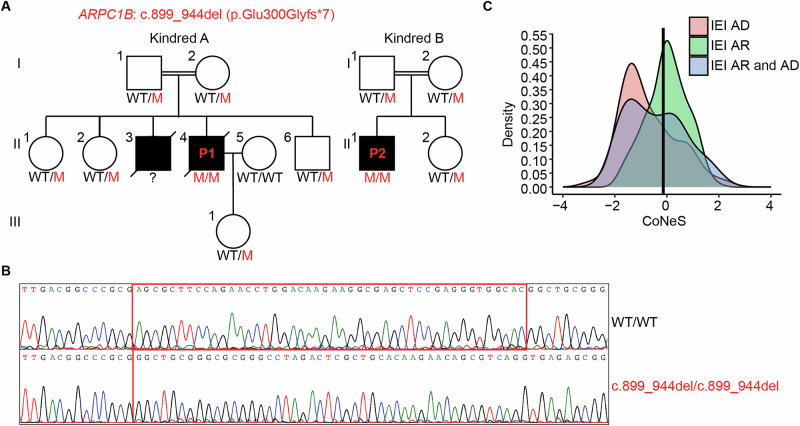
c.899_944del mutation in *ARPC1B.* **A** Familial segregation of the mutation in two unrelated families. WT: wild-type, M: mutant. **B** Electropherogram of a healthy control and a patient (P1) at the site of the c.899_944del mutation on Exon 8 of *ARPC1B*. **C** Distribution of Consensus Negative Selection (CoNeS) scores for genes causing Inborn Errors of Immunity (IEI) when mutated. *ARPC1B* is indicated with a vertical line. AD autosomal dominant, AR autosomal recessive.

**Fig. 2:**
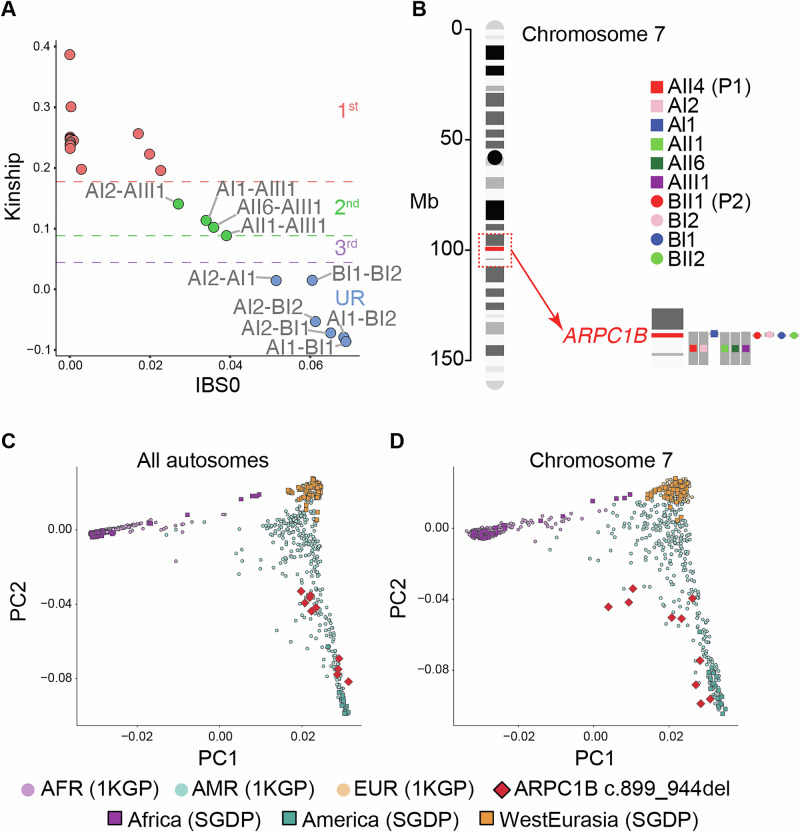
Age of the c.899_944del variant and genetic ancestry of variant carriers. **A** Scatter plot showing pairwise relatedness inferred using KING, with kinship coefficient (y-axis) and IBS0 (proportion of variants with zero alleles identical by state, x-axis). Each point represents an individual pair. Colors indicate relationship categories defined by kinship coefficient thresholds of KING: first-degree (red), second-degree (green), third-degree (purple), and unrelated pairs (UR, blue). **B** Shared haplotype block identified in the haplotype analysis of the chromosome 7 region surrounding the c.899_944del variant. **C** Principal Component Analysis (PCA) of genetic ancestry for c.899_944del carriers based on all autosomes. **D** PCA of genetic ancestry using chromosome 7 variants.

We describe two unrelated patients from Mexico, both coming from consanguineous parents, who presented with similar clinical symptoms of CID (Fig. 1A and Supplementary Material). Genetic testing showed that both have the same previously reported homozygous mutation in *ARPC1B* (c.899_944del, p.E300Gfs*7) (Fig. 1B) [6, 9]. This mutation segregates with the disease as an autosomal recessive trait (Fig. 1A). The Consensus-Based Measure of Negative Selection (CoNeS) score shows that *ARPC1B* aligns with genes known to cause inborn errors of immunity inherited in an autosomal recessive manner (Fig. 1C). The mutation is present in the gnomAD database with a minor allele frequency of 6.453e–7 corresponding to one heterozygous carrier. The geographical proximity of these two families, along with the autosomal recessive inheritance of this mutation, prompted an investigation into its origin. To study this, we performed whole genome sequencing. Using kinship analysis, we confirmed the presence of first- and second-degree relatives within pedigrees, and the absence of third-degree or closer relationships between the parents and between the two families (Fig. 2A). By haplotype analysis on the region of Chromosome 7 that contains *ARPC1B*, we identified a conserved haplotype segment around the mutation site in all patients and family members (Fig. 2B). The length of the shared haplotype segment demonstrates that the two patients shared a common ancestor approximately 55 generations ago (95% confidence interval, 10.2–105.8). The presence of a common ancestor indicates that the c.899_944del variant in *ARPC1B* originated through a founder effect. We investigated the ancestral origin of the allele to confirm if this variant is native to the indigenous American population. We assessed the genetic ancestry of the ten variant carriers using a combined dataset from the 1KGP and SGDP. By ancestry inference analysis conducted on the whole genome (Fig. 2C) and chromosome 7 (Fig. 2D), we showed that the carriers of the c.899_944del mutation cluster with Amerindian (1KGP) and indigenous American (SGDP) populations, indicating that this mutation arose in the native American population. Together, these results indicate that the c.899_944del variant of the *ARPC1B* gene emerged through a founder effect in the indigenous American population.

### The ARPC1B p.E300Gfs*7 mutation alters protein expression

**Fig. 3:**
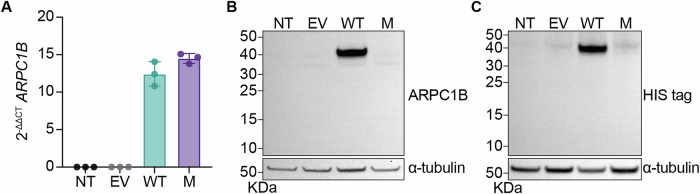
p.E300Gfs*7 mutation leads to loss of expression of ARPC1B. **A** RT-qPCR results showing the *ARPC1B* mRNA levels for HEK293T cells transfected with empty vector (EV), wild-type (WT) or mutant (M) plasmids or non-transfected (NT) cells. *GUS* was used as a control for gene expression. Error bars represent SD for three technical replicates. Graph is representative of three independent experiments. Western blots of lysates from the transfected HEK293T cells shown in A showing expression of (**B**) ARPC1B and (**C**) His tag. α-tubulin was used as a loading control. Observed molecular weight of ARPC1B is 38 kDa. Each western blot is representative of three individual experiments.

The mutation found in our patients (c.899_944del) causes the deletion of 46 base pairs in exon 8 of the gene (Fig. 1B). At the protein level, ARPC1B contains six WD40 domains that form a β-propeller [4], and the mutation locates between the 5th and 6th WD40 domains. This deletion is predicted to cause a shift in the reading frame, introducing a six amino acid aberrant sequence (GLRARA) followed by a premature stop codon. In order to analyze the effects of this mutation on mRNA and protein expression, we overexpressed WT or p.E300Gfs*7 ARPC1B with a C- terminal His-tag in HEK293T cells. We did not observe any significant difference in *ARPC1B* mRNA levels between the WT and mutant, suggesting that the mutation does not lead to nonsense-mediated mRNA decay (Fig. 3A). Using western blotting, we did not observe ARPC1B expression for the mutant with a polyclonal anti-ARPC1B antibody made with an immunogen that contains an amino acid sequence predominately N terminal of the mutation. This suggests that, with the available approach, the expression of the p.E300Gfs*7 mutant allele is not detectable (Fig. 3B). Furthermore, using an anti-His tag antibody, we again could not detect ARPC1B expression in the mutant, confirming that there is no reinitiation of translation after the premature stop codon caused by the mutation (Fig. 3C). These results indicate that the p.E300Gfs*7 mutation likely leads to complete loss of ARPC1B expression.

### T cell lymphopenia and increased memory vs. naïve ratio in ARPC1B deficiency

**Fig. 4:**
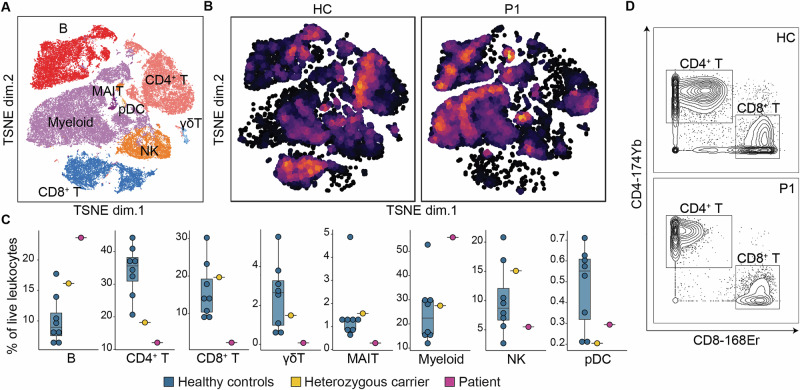
Immunophenotyping by CyTOF. **A** Dimensional reduction by t-SNE of 33 surface markers. Each color identifies a distinct cell population. **B** Density t-SNE plots of healthy controls (HC) and the patient (P1). **C** Box plot representing maximum and minimum values, interquartile range, and the median of the frequencies of immune cell populations as a percentage of total leukocytes for healthy controls, a heterozygous carrier, and the patient. **D** Gating of the CD4^+^ and CD8^+^ T cell populations in a healthy control (HC) and the patient (P1). Parental gating: CD45^+^ CD66b^-^ CD19^-^ CD20^-^ CD14^-^ CD56^-^ CD3^+^.

**Fig. 5:**
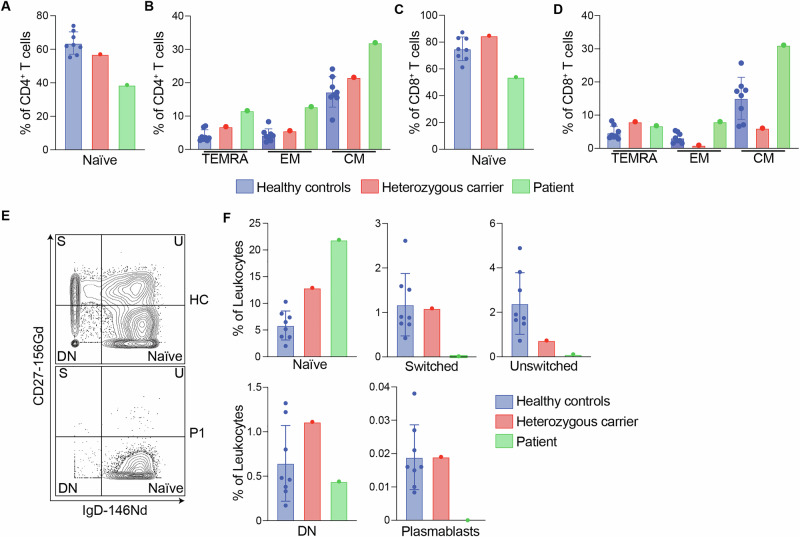
Frequencies analysis of naïve and memory B and T cells. **A** Frequencies of naïve CD4^+^ T cells as a percentage of total CD4^+^ T cells in healthy controls, a heterozygous carrier and the patient. The error bars represent the SD. **B** CD4^+^ Terminal Effector Memory Re-expressing CD45RA (TEMRA), Effector Memory (EM), and Central Memory (CM) T cell frequencies as a percentage of total CD4^+^ T cells. The error bars represent the SD. **C** Frequencies of naïve CD8^+^ T cells as a percentage of total CD8^+^ T cells. **D** CD8^+^ Terminal Effector Memory Re-expressing CD45RA (TEMRA), Effector Memory (EM), and Central Memory (CM) T cell frequencies as a percentage of total CD8^+^ T cells. The error bars represent the SD. **E** Example of manual gating of CD27 vs IgD for healthy control (HC) and patient (P1) CD19^+^CD20^+^ B cells. Parental gating: CD45^+^ CD66b^-^ CD19^+^ CD20^+^. S: switched, U: unswitched DN: double-negative. **F** Frequencies of B cell subsets as a percentage of total leukocytes in healthy controls, a heterozygous carrier, and the patient. The error bars represent the SD.

To better understand the effect that ARPC1B deficiency has on the overall makeup of the immune system, we carried out in-depth immunophenotyping using CyTOF on PBMCs from P1, his heterozygous father, and healthy controls. We used a 33-antibody panel (Table S2) [31] against surface markers of common leukocyte populations. By unsupervised clustering, we were able to determine eight distinct immune cell populations– B cells, CD4^+^ T cells, CD8^+^ T cells, γδ T cells, mucosal-associated invariant T cells (MAIT), myeloid cells, plasmacytoid dendritic cells (pDC), and natural killer (NK) cells (Fig. 4A, B and S2). We observed a reduction in the frequencies of total CD4^+^, CD8^+^, and γδ T cells in our patient compared to healthy controls and the heterozygous carrier (Fig. 4C, D and Table S3). To further characterize this phenotype, we analyzed the populations of CD4^+^ and CD8^+^ cells individually to determine the frequencies of their subset compositions. We used manual gating with surface marker expression to categorize the subsets of CD4^+^ and CD8^+^ cells as central memory, effector memory, naïve, and terminal effector memory re-expressing CD45RA (TEMRA) T cells (Fig. S1). The frequencies of these subsets revealed that the patient had a strikingly low number of naïve CD4^+^ and CD8^+^ T cells compared to healthy controls and the heterozygous carrier. We also found that P1 had an increased percentage of both CD4^+^ and CD8^+^ central memory and effector memory cells compared to controls, as well as increased CD4^+^ TEMRA cells (Fig. 5A–D). Overall, our results suggest that ARPC1B deficiency greatly impacts T cell development.

### ARPC1B deficient B cells show reduced frequencies of switched and unswitched cells

In addition to the reduced T cell frequencies described above, we observed that the cell distributions within B cell compartments differed between healthy controls and the patient (Fig. 4B). To better characterize this observation, we performed manual gating which showed that the switched and unswitched memory B cell populations, as well as plasmablasts, were drastically reduced in the patient in comparison to the healthy controls (Fig. 5E, F). These results suggest that ARPC1B deficiency may impair class-switching in B cells.

### Clinical and immunological spectrum of ARPC1B deficiency

To place patients with the mutation p.E300Gfs*7 in context with other ARPC1B deficient patients, we summarized the clinical symptoms and immunological consequences of each reported ARPC1B deficient patient (Table 1 and S4). Clinically, our two patients share similar symptoms with many of the other ARPC1B patients listed in the table as common symptoms include eczema and recurrent infections such as pneumonia. While other ARPC1B patients have experienced skin lesions as a result of dermatitis, both of our patients formed keloid scars from their wounds which are not present in most other patients. Additionally, our patients did not have food allergies, which is a common symptom of ARPC1B deficiency. Of the 37 patients that have immunological cell count data previously reported, approximately 40% had high B cell counts, and 10 patients were found to have low naïve CD4^+^ T cells, showing similar characteristics to our patient. However, while a few other patients had high memory CD4^+^ T cells, consistent with what we found in P1 with our CyTOF data, 5 patients were reported to have an abnormally low memory T cell subset. Overall, this summary highlights the clinical and immunological heterogeneity that ARPC1B deficiency can cause.

**Table 1:** Table describing the main clinical, genetic and immunological findings of all the previously reported ARPC1B deficient patients.

Reference	Origin	Clinical Symptoms	Variant	Amino Acid Consequence	Zygosity	Immunological Findings
Kuijpers et al. [5], Volpi et al. [41] (P1)	Morocco	Gastric bleeding, eczema, food allergy, failure to thrive, diarrhea, hepatomegaly, leukocytoclastic vasculitis, infections such as gastroenteritis, auricular infection, and recurrent pneumonia	491_495delinsCCTGCCC	p.Phe164SerfsTer31	Homozygous	Increased IgE and IgA, low naïve CD4+ and CD8 + T cells, increased number of B cells
Brigida et al. [36] (P1), Volpi et al. [41] (P2)	Italy	Recurring hemorrhagic enterocolitis, failure to thrive, eczema, leukocytoclastic vasculitis, recurrent pneumonia, warts on hands and face	c.64+1 G > C	NA	Homozygous	Increased IgE and IgA, low naïve CD4+ and CD8 + T cells, low NK cells, increased number of B cells
Brigida et al. [36] (P2), Volpi et al. [41](P3)	Italy	Fevers, polymorphic vasulitic rash, hematochezia, hepatosplenomegaly, eczema, failure to thrive, leukocytoclastic vasculitis, lymphodenopathy, infections such as CMV, recurrent otitis,	622 G > T	p.Val208Phe	Homozygous	Increased IgE and IgA, low naïve CD4+ and CD8 + T cells, increased number of B cells
Brigida et al. [36] (P3), Yan et al. [42]	Canada	Eczema, food allergies, diarrhea, failure to thrive, lymphadenopathy, hepatosplenomegaly, infections such as otitis media, severe balanitis, chickenpox, perianal area infections, pansinusitis, warts	c.1087dup	p.Glu363GlyfsTer95	Homozygous	high IgE and IgA, reduced naïve and memory CD4 + T cells
Brigida et al. [36] (P4)	Colombia	Gluteal and pulmonary abscesses, eczema, diarrhea, failure to thrive, lymphadenopathy, hepatosplenomegaly, inflammatory colitis, gastointestinal bleeding, gingival, hemorrhagic enteritis, infections such as recurrent pneumonia, bronchiolitis, sepsis	c.258 G > A	p.Trp86Ter	Homozygous	High IgE, slightly increased B cell count, reduced CD4+ and CD8+ naïve, high CD4+ and CD8+ memory, high NK cell count
Brigida et al. [36] (P5)	Morocco	Eczema, diarrhea, oral thrush, infections such as recurrent pneumonia and a cold abcess in lung	c.318del	p.Asn107ThrfsTer13	Homozygous	High IgE, high B cell count, low CD4+ and CD8+ naïve, high CD4+ memory
Brigida et al. [36] (P6)	Turkey	Eczema, diarrhea, failure to thrive, lymphadenopathy, hepatosplenomegaly, keratotic hair lesions, psychomotor retardation,perianal and vulvar necrotizing ulcerations, infections such as lung and joint abcesses, bacterial meningitis, recurrent otorrhea	c.64+1 G > A	NA	Homozygous	High IgE and IgA, low CD4+ and CD8+ naïve T cells
Somech et al. [35] (P1)	Israel	Eczema, colitis, severe thrombocytopenia, infections such as perianal abscess, lymphadenitis, recurrent pneumonia	c.G623del-TC	p.Val208ValfsTer20	Homozygous	Increased B cells, high IgG and IgE levels, reduced CD3 + , reduced CD8 + T cells
Brigida et al. [36](P7), Somech et al. [35] (P2)	Isreal	Eczema, diarrhea, fever, otitis media, thrombocytopenia	c.G623del-TC	p.Val208ValfsTer20	Homozygous	Increased B cells, high IgG and IgE levels, reduced CD3 + , reduced CD8 + T cells
Kahr et al. [4] (P1), Volpi et al. [41] (P9)	South Asia	Eczema, diarrhea, failure to thrive, lymphadenopathy, leukocytoclastic vasculitis, skeletal abnormalities, thrombocytopenia, infections such as meningitis, septic shock, pneumonia, gastroenteritis, multiple abscesses	c.269_270dupCT	p.Val91TrpfsTer30	Homozygous	High IgA and IgE, high B cell count, low CD8 + T cells
Kahr et al. [4] (P2), Volpi et al. [41] (P10)	Scotland	Eczema, failure to thrive, leukocytoclastic vasculitis, subconjunctival hemorrhage, brain vasculitis	c.314 C > T	p.Ala105Val	Homozygous	High IgA and IgE, high B cell count
Kahr et al. [4] (P3), Volpi et al. [41] (P11)	Scotland	Eczema, skin lesions, asthma, failure to thrive, infections such as pneumonia and impetigo of the face	c.314 C > T	p.Ala105Val	Homozygous	High IgA and IgE, high B cell count
Volpi et al. [41] (P4)	Nepal	Eczema, asthma, failure to thrive, stridor due to tracheomalacia, hematemesis with wheezing, infections such as bronchiolitis and otitis media	c.64+2 T > A	NA	Homozygous	High IgE, IgG, and IgA, low naïve CD4 + T cells, low CD8 + T cells, increased number of B cells
Volpi et al. [41] (P5)	Somalia	Eczema, diarrhea, failure to thrive, skin abscesses, warts, infections such as mastoiditis, otitis media, periodontitis, CMV viremia, and pneumonia	c.392+2 T > C	NA	Homozygous	Low naïve CD4 + T cells
Volpi et al. [41] (P6)	Morocco	Eczema, diarrhea, failure to thrive, skin abscess, gastroenteritis, recurrent infections such as bronchitis, pneumonia, and otitis media	c.311 G > C	p.Trp104Ser	Homozygous	High IgE and IgA, low naïve CD4 + , low CD8 + T cells, increased number of B cells
Volpi et al. [41] (P7)	Iran	Generalized maculopapular rash, eczema,food allergies, allergy to dust, failure to thrive, severe skin vasculitis, infections such as perianal abscess, fungal otitis externa, ecthyma gangrenosum, and recurrent pneumonia	c.897_910delCGAGCGCTTCCAGA	p.Glu300ProfsTer153	Homozygous	High IgA and IgE, high CD8+ cell count, high NK cell count, elevated B cell count
Volpi et al. [41] (P8)	Iran	Generalized maculopapular rash, eczema, food allergies, failure to thrive, rectorrhagia, severe eosinophilic colitis, infections such as meningitis, otitis media,purulent axillary lymphodenitis, recurrent pneumonia	c.897_910delCGAGCGCTTCCAGA	p.Glu300ProfsTer153	Homozygous	High IgE and IgA, igh NK cell count
Volpi et al. [41] (P12)	Nepal	Erosize dermatitis, ulcerative lesions, eczema, food allergies, oral thrush, failure to thrive, diarrhea, perianal ulcer, leukocytoclastic vasculitis, infections such as CMV, UTI, otitis media periorbital cellulitis	c.64+2 T > A	NA	Homozygous	High IgM, IgG, IgA, and IgE
Volpi et al. [41] (P13)	Jordan	Leukocytoclastic vasculitis, chronic arthritis, recurrent infections such as otitis media and pneumonia	c.708-1 G > A	NA	Homozygous	High IgA and IgE, low CD4 + T cells, low B cell count
Vopli et al. [41] (P14)	Nepal	Eczema, anemia, failure to thrive, thrombocytopenia, lymphoadenopathy with microcytic anemia, hemoptysis, enterorrhagia, polyarthritis, and recurrent infections such as otitis media, periodontal disease, and bronchiolitis	c.64+2 T > A	NA	Homozygous	High IgE and IgA
Papadatou et al. [43] (P1 or II-6)	Afghanistan	Eczema, food allergies, asthma, failure to thrive, autoimmune hypothyroidism, perianal and skin abscesses, infections such as pneumonia and bronchiolitis	c.783 G > A	p.Ala261Ala (loss of donor splice site)	Homozygous	High IgA, low CD3+ count, high naïve/memory CD4+ ratio
Papadatou et al. [43] (P2 or II-1)	Afghanistan	Eczema, failure to thrive, lymphadenopathy, skin abscesses, hypothyroidism, recurrent pneumonia	c.783 G > A	p.Ala261Ala (loss of donor splice site)	Homozygous	High IgE, IgG,and IgA, high naïve/memory CD4+ ratio
Papadatou et al. [43] (P3 or II-3)	Afghanistan	Autoimmune hypothyroidism, failure to thrive, recurrent viral upper respiratory tract infections	c.783 G > A	p.Ala261Ala (loss of donor splice site)	Homozygous	High IgE and IgA count, high naïve/memory CD4+ ratio
Kopitar et al. [44] (P1)	Slovenia	Eczema, thrombocytopenia, diarrhea, food allergies, pollen allergy, failure to thrive, enterocolitis, small vessels vasculitis, infections such as pneuomonia, bronchiolitis, panniculitis, gastroenteritis	c.265 A > C	p.Thr89Pro	Homozygous	High IgE, low CD3+ and CD4 + , low T cell proliferation, high CD19+
Kopitar et al. [44] (P2)	Slovenia	Eczema, thrombocytopenia, diarrhea, skin abscesses, food allergies, failure to thrive, autoimmune vasculitis, warts, gastroenteritis, enterocolitis, pneuomonia	c.265 A > C	p.Thr89Pro	Homozygous	High IgE, high CD19 + , low T cell proliferation
Kopitar et al. [44] (P3)	Slovenia	Eczema, thrombocytopenia, diarrhea, food allergies, animal epithelia allergy, asthma failure to thrive, small vessels vasculitis, autoimmune vasculitis, warts, enterocolitis, skin abscesses, recurrent pneumonia	c.265 A > C	p.Thr89Pro	Homozygous	High IgE and I gA, low CD8 + , low NK,
Kopitar et al. [44] (P4)	Slovenia	Eczema, Evans syndrome	c.265 A > C	p.Thr89Pro	Homozygous	High IgG and IgA, low CD3 + , low CD4+ and CD8+
Chiriaco et al. [8]		Diarrhea, perianal and oral ulcers, eosinophilia, thrombocytopeni, severe atopic dermatitis, sepsis, recurrent otitis	c.212_226del	p.Gly71_Asn75del	Homozygous	Low memory B cell subset, increased regulatory T cells, increased memory T cell subset
Vásquez-Echeverri et al. [6] (P1)	Mexico	Eczema, diarrhea, food allergies, failure to thrive, lymphadenopathy, thrombocytopenia, hepatosplenomegaly, infections such as UTI, tuburculosis, sepsis, recurrent pneumonia, rectal prolapse and rectovescial fistula	c.899_944del	p.Glu300GlyfsTer31	Homozygous	Hyper- IgE, high IgG, high IgA, high IgM, low serum complement (C3 and C4), low CD8+ cell count
Vásquez-Echeverri et al. [6] (P2)	Mexico	Eczema, rectal bleeding, food allergies, ulcer, infections such as oral candidiasis, bronchiolitis, and pneumonia	c.899_944del	p.Glu300GlyfsTer31	Homozygous	High IgG and IgA, low CD8 + , high CD19+ and CD16/56+
Vásquez-Echeverri et al. [6] (P3)	Mexico	Eczema, failure to thrive, hematochezia, heptaomegaly, diarrhea, infections such as bronchiolitis, orbital cellulitis, sepsis, recurrent pneumonia and gastroenteritis	c.64 C > T/c.899_944del	p.Gln22Ter/p.Glu300GlyfsTer31	Compound heterozygous	High IgG, IgA and IgE, high CD19 + , low CD4+ and CD8 + , high CD56 + /16+
Vásquez-Echeverri et al. [6] (P5)	Mexico	Epistaxis, ecchymoses, gum bleeding, jaundice, lymphadenopathy, hepatosplenomegaly, infections such as chronic viral hepatitis	c.94 G > A/c.111 G > C	p.Val32Met/Lys37Asn	Compound heterozygous	High IgG, IgA and IgE, very low B cell count
Vásquez-Echeverri et al. [6] (P6), our Patient 1	Mexico	Anal fistula, eczema, keloids, skin and abdominal vastulitis, warts, infections such as UTI, pneumonia, gastroenteritis	c.899_944del	p.Glu300GlyfsTer31	Homozygous	High IgA, IgM, and IgE, low IgG, low CD4 + , low CD8 + , high B cell count
Zavaleta Martinez et al. [9] (P1), our Patient 2	Mexico	Eczema, allergies, skin abscesses, keloid scarring, pneumonia, anemia, thrombocytopenia,	c.899_944del	p.Glu300GlyfsTer31	Homozygous	High IgA and IgE, high CD56 + /16 + , low CD4+ and CD8 + , low C3 and C4
Zavaleta Martinez et al. [9] (P2)		Eczema, food allergies, facial cellulitis, anemia, thrombotcytopenia, chronic external otitis, fever, abscess on thorax, erythematous, scaly dermatitis	c.863del	p.Pro288Leufs*9	Homozygous	High IgG, IgM, IgA, and IgE, high CD19 + , high CD56 + /16+
Sonmez et al. [7]		Diarrhea, food allergies, recurrent fever, skin lesions, eosinophilia, oral thrush, skin rash and lesions,	c.1081-5 T > G	NA	Homozygous	Increased naïve and decreased switched B cells, decreased naïve CD8 + T cells, CD8 + EM T cells, decreased CD4+ central memory T cells, increased CD4+ and CD8 + TEMRA

## Discussion

In this study, we characterized the c.899_944del *ARPC1B* variant as a mutation that arose due to a founder effect. This mutation was first described by Vásquez-Echeverri et al. in 2023 [6]. As the variant was present in three unrelated families living in distinct regions of Mexico, the authors hypothesized that it may have originated through a founder effect. In our haplotype and ancestry inference analysis, we confirmed this hypothesis since we were able to identify this 46-base pair deletion as emerging 55 generations ago in the indigenous American population (Fig. 2A–D). Our findings also showed that this variant leads to complete deficiency of ARPC1B protein expression (Fig. 3B, C). ARPC1B has been shown to be an imperative protein for filamentous actin (F-actin) polymerization in immune cells [32]. Deficiency in ARPC1B disrupts this process leading to devastating effects on many cellular functions including cell migration and adhesion, endocytosis, phagocytosis, and mitosis [33]. These effects can manifest in a wide range of clinical symptoms indicative of CID, which are outlined in Table 1 and include severe, fatal infection. Previous patients with ARPC1B deficiency, including P2, have responded well to hematopoietic stem cell transplantation [34]. However, prompt diagnosis is important. Diagnosing ARPC1B deficiency can be challenging due to the rarity of the disease, genetic database limitations, and the wide range of CID symptoms. The findings from our study could allow for targeted genetic testing and quicker diagnosis and treatment for patients presenting CID symptoms consistent with ARPC1B deficiency, especially in Mexico.

While there have been previous studies investigating how ARPC1B deficiency impacts individual cell populations, our report is the first that has carried out in-depth immunophenotyping using CyTOF to analyze the frequencies of various subsets of immune cells in an ARPC1B-deficient patient compared to healthy controls. Through this analysis, we were able to gain a better understanding of how ARPC1B deficiency impacts the overall structure of the immune system as well as how it impacts specific subpopulations of immune cell types. Previous studies have shown that ARPC1B deficiency has a detrimental impact on the ability of T cells to function as ARPC1B is important for T cell receptor activation and immunological synapse formation [35, 36]. Our mass cytometry data revealed a decreased frequency of total T cells in our patient compared to healthy controls (Fig. 4B–D), which aligns with case reports that note low total T cell counts in ARPC1B patients (Table 1 and S4). We further analyzed the frequencies of the CD4^+^ and CD8^+^ T cell subsets as percentages of CD4^+^ or CD8^+^ T cells. Our analysis revealed that our patient had diminished amounts of CD4^+^ and CD8^+^ naïve T cells and elevated frequencies of CD4^+^ and CD8^+^ effector memory and central memory T cells within the CD4^+^ and CD8^+^ T cell compartments (Fig. 5A–D). This data displays an abnormal ratio of naïve/memory T cells in our patient in both the CD4^+^ and CD8^+^ cell compartments. A low naïve/memory T cell ratio is an indicator of other IEI that are known to impact the function of the thymus [37]. While a previous report has suggested that ARPC1B deficiency can also hinder thymic output [35], future investigation needs to be done to understand the mechanisms that cause this disruption in ARPC1B deficient T cells.

As we continued to analyze the subsets of the immune cell populations, we found that our patient had very few class-switched B cells (Fig. 5E, F). Actin polymerization is required for the B cell signaling process as this process involves the rapid clustering of B cell receptors (BCRs) [38]. In steady state, BCRs are spatially compartmentalized by F-actin anchored to the plasma membrane. When a BCR encounters an antigen, the F-actin compartments disassemble to allow for BCRs to increase mobility and form clusters. F-actin must then reassemble at the edge of the cell to increase the antigen-contacting surface for further engagement of clustering BCRs [39]. As ARPC1B is a critical protein for actin polymerization, it is likely involved in many of these steps, though its role has not been elucidated. It has been found previously that ARPC1B is important for regulating tonic signaling by supporting the steady state cytoskeleton [32], however, the mechanism and extent to which ARPC1B is involved is unknown. Nevertheless, as both antigen-induced and tonic B cell signaling are necessary for B cell maturation, ARPC1B’s role in actin polymerization can help explain the lack of class-switched B cells in our patient. However, it is also possible that this could be a secondary effect to the abnormally low frequency of T cells in our patient. Some CID-causing IEIs lead to diminished class-switched B cells because T follicular helper (Tfh) cell counts are affected. An example of this is inducible T cell co-stimulator (ICOS) deficiency, in which the loss of an important protein in T cell activation leads to similar B cell maturation affects as seen in our ARPC1B-deficient patient [40]. While the study by Leung and colleagues [32] demonstrates that ARPC1B plays some role in B cell signaling, it is unclear as to how much of the class-switched B cell deficiency seen in our patient is attributed to a B cell intrinsic consequence of the variant rather than a compounded affect from the low amount of T cells present. More studies should be done to isolate ARPC1B-deficient B cells and test the effects on their function and development.

There is still more work that needs to be done to fully understand how ARPC1B fits into overall immune function. Despite the detailed insights we obtained about the immunological consequences of ARPC1B deficiency, it is worth noting that our conclusions were obtained from the study of a single patient. Additionally, our experiments were done with cryopreserved PBMCs, so we were unable to obtain an absolute cell count. Gathering data from more individuals with ARPC1B deficiency in the future would allow for a clearer overview of the immunological effects of this disease. Nevertheless, our immunophenotyping results give insight into how widespread ARPC1B’s role is in the immune system, as it is clear that the absence of this protein has drastic impacts across many different immune cell types.

In conclusion, this study characterized the c.899_944del variant in *ARPC1B* as a founder mutation of indigenous American ancestry that leads to loss of protein expression. This knowledge can assist in quicker diagnosis in patients from Mexico presenting with CID symptoms and allow for life-saving treatment. Additionally, complete immunophenotyping data from an ARPC1B-deficient patient displays the vast impact that this deficiency can have on the immune system. The data obtained from CyTOF analysis revealed that ARPC1B deficiency disrupts several immune cell compartments including class-switched memory B cells, total T cells and memory T cells. These results help build the knowledge of this rare disease and add to the context of the intricate role that ARPC1B performs in the immune response.

## Supplementary information

Supplementary table 1

Supplementary table 2

Supplementary table 3

Supplementary table 4

Supplementary material

## Data Availability

The data generated during this study is available from the corresponding author upon reasonable request.
