# Supplementary material for: Molecular analysis and immunological characterization of a founder mutation causing ARPC1B deficiency
Source: Genes Immun. 2025 Nov 17;27(1):69–80. doi: 10.1038/s41435-025-00368-w (PMC12923354; doi:10.1038/s41435-025-00368-w)
Supplement: Supplementary file 1 — Supplementary table 1 [file 41435_2025_368_MOESM1_ESM.docx]

**Supplementary table 1: Primers**

| **Primers** | **Sequence** |
| --- | --- |
| Amplification from genomic DNA and sequencing to confirm mutation- Forward | GTTTTGATACACCTGCAATTC |
| Amplification from genomic DNA and sequencing to confirm mutation- Reverse | CCATCTGATGCTATTTTTATGC |
| C terminal His Tag WT- Reverse | TCAGTGGTGATGGTGATGATGTTTGATCTTGAGGTCCTTCA |
| C terminal His Tag M- Forward | CACCATGGCCTACCACAGCT |
| C terminal His Tag M- Reverse | TCAGTGGTGATGGTGATGATGTTTGATCTTGAGGTCCTTCA |
| Sequencing Primer 1 | ATGGCCTACCACAGCT |
| Sequencing Primer 2 | AAGGGCCGCACATGGA |
| Sequencing Primer 3 | GTGGGGCTCCAAGATG |
| Sequencing Primer 4 | GCTGGACGTTCCTAAG |
| Sequencing Primer 5 | CAAGGCCAAGTGCTCG |
| Sequencing Primer 6 | TTGTTCCAGGCGTGGC |
| T7 Primer | TAATACGACTCACTATAGGG |
| BGH Primer | TAGAAGGCACAGTCGAGG |
| ARPC1B qPCR- Forward | TGACTGGTGGGTTTGCAA |
| ARPC1B qPCR- Reverse | CTGAAAAGATCCGACACTT |
| GUS qPCR- Forward | CTACTTGAAGATGGTGATCG |
| GUS qPCR- Reverse | CTGTTCAAACAGATCACATC |
| C terminal His Tag WT- Forward | ATGGCCTACCACAGCT |
