# Supplementary material for: Molecular analysis and immunological characterization of a founder mutation causing ARPC1B deficiency
Source: Genes Immun. 2025 Nov 17;27(1):69–80. doi: 10.1038/s41435-025-00368-w (PMC12923354; doi:10.1038/s41435-025-00368-w)
Supplement: Supplementary file 2 — Supplementary table 2 [file 41435_2025_368_MOESM2_ESM.docx]

**Supplementary Table 2. Antibodies used for mass cytometry.**

| Antibody | Clone | Isotype | Dilution | Company |
| --- | --- | --- | --- | --- |
| Anti-Human CD45 | H130 | 89Y | 1:50 | Standard BioTools |
| Anti-Human CD19 | HIB19 | 142Nd | 1:50 | Standard BioTools |
| Anti-Human CD127/IL7Ra | A019D5 | 143Nd | 1:50 | Standard BioTools |
| Anti-Human CD38 | HIT2 | 144Nd | 1:50 | Standard BioTools |
| Anti-Human IgD | IA6-2 | 146Nd | 1:50 | Standard BioTools |
| Anti-Human CD11c | Bu15 | 147Sm | 1:50 | Standard BioTools |
| Anti-Human CD16 | 3G8 | 148Nd | 1:50 | Standard BioTools |
| Anti-Human CD194/CCR4 | L291H4 | 149Sm | 1:50 | Standard BioTools |
| Anti-Human CD123/IL-3R | 6H6 | 151Eu | 1:50 | Standard BioTools |
| Anti-Human TCRgd | 11F2 | 152Sm | 1:50 | Standard BioTools |
| Anti-Human CD185/CXCR5 | RF8B2 | 153Eu | 1:50 | Standard BioTools |
| Anti-Human CD3 | UCHT1 | 154Sm | 1:50 | Standard BioTools |
| Anti-Human CD45RA | HI100 | 155Gd | 1:50 | Standard BioTools |
| Anti-Human CD27 | L128 | 158Gd | 1:50 | Standard BioTools |
| Anti-Human CD28 | CD28.2 | 160Gd | 1:50 | Standard BioTools |
| Anti-Human CD66b | 80H3 | 162Dy | 1:50 | Standard BioTools |
| Anti-Human CD183/CXCR3 | G025H7 | 163Dy | 1:50 | Standard BioTools |
| Anti-Human CD161 | HP-3G10 | 164Dy | 1:50 | Standard BioTools |
| Anti-Human CD45RO | UCHL1 | 165Ho | 1:50 | Standard BioTools |
| Anti-Human CD24 | ML5 | 166Er | 1:50 | Standard BioTools |
| Anti-Human CD197/CCR7 | G043H7 | 167Er | 1:50 | Standard BioTools |
| Anti-Human CD8 | SK1 | 168Er | 1:50 | Standard BioTools |
| Anti-Human CD25 | 2A3 | 169Tm | 1:50 | Standard BioTools |
| Anti-Human CD20 | 2H7 | 171Yb | 1:50 | Standard BioTools |
| Anti-Human HLA-DR | L243 | 173Yb | 1:50 | Standard BioTools |
| Anti-Human CD4 | SK3 | 174Yb | 1:50 | Standard BioTools |
| Anti-Human CD56 | NCAM16.2 | 176Yb | 1:50 | Standard BioTools |
| Anti-Human CD196 | G034E3 | 141Pr | 1:25 | Standard BioTools |
| Anti-Human CD14 | M5E2 | 175Lu | 1:25 | Standard BioTools |
| Anti-Human CD117 | 104D2 | 150Nd | 1:25 | Biolegend |
| Anti-Human TCR Vα24/Jα18 | 6B11 | 156Gb | 1:25 | Biolegend |
| Anti-Human TCR Vα7.2 | 3C10 | 159Tb | 1:25 | Biolegend |
| Anti-Human CD294 | BM16 | 161Dy | 1:25 | Biolegend |
