# Supplementary material for: Molecular analysis and immunological characterization of a founder mutation causing ARPC1B deficiency
Source: Genes Immun. 2025 Nov 17;27(1):69–80. doi: 10.1038/s41435-025-00368-w (PMC12923354; doi:10.1038/s41435-025-00368-w)
Supplement: Supplementary file 3 — Supplementary table 3 [file 41435_2025_368_MOESM3_ESM.docx]

**Supplementary Table 3: Cell Frequencies obtained by CyTOF.**

| **Population as percentage of Live Leukocytes** | **Range of Healthy Controls** | **Value in Patient** |
| --- | --- | --- |
| B Cells | 6.39 - 17.1 | 22.5 |
| Switched B Cells | 0.42 - 2.61 | 0.014 |
| Unswitched B Cells | 0.72 - 4.89 | 0.11 |
| Naïve B Cells | 2.02 - 10.3 | 21.9 |
| DN B Cells | 0.17 - 1.32 | 0.44 |
| Plasmablasts | 8.36E-03 - 0.038 | 0 |
| Transitional B Cells | 0.052 - 0.14 | 0.38 |
| CD4+ T Cells | 13.1 - 35.8 | 11.6 |
| T Regs | 0.33 - 1.53 | 0.48 |
| Follicular T Cells | 0.033 - 3.18 | 0.042 |
| CD4^+^ TEMRA | 0.58 - 1.94 | 1.35 |
| CD4^+^ Naïve | 9.1 - 22.4 | 4.49 |
| CD4^+^ Central Memory | 1.16 - 7.52 | 3.71 |
| CD4^+^ Effector Memory | 0.29 - 2.28 | 1.49 |
| Th1 | 0.29 - 1.56 | 0.2 |
| Th2 | 0.35 - 1.93 | 1.65 |
| Th17 | 0.22 - 1.26 | 2.2 |
| Th1* | 0.067 - 0.81 | 0.23 |
| CD8^+^ T Cells | 6.03 - 26.3 | 2.12 |
| CD8^+^TEMRA | 0.31 - 1.28 | 0.14 |
| CD8^+^ Naïve | 3.69 - 21.8 | 1.14 |
| CD8^+^ Central Memory | 1.08 - 3.17 | 0.66 |
| CD8^+^ Effector Memory | 0.19 - 0.57 | 0.17 |
| NK Cells | 3.73 - 20.5 | 7.64 |
| NK CD56br | 5.58E-03 - 0.41 | 0.18 |
| NK CD56dimCD16^+^ | 1.72 - 15.1 | 3.17 |
| NK CD56dimCD56^-^ | 0.56 - 5.18 | 4.24 |
| Non-Classical Monocytes | 0.37 - 6.25 | 6.66 |
| Intermediate Monocytes | 0.035 - 3.15 | 3.52 |
| Classical Monocytes | 1.49 - 26.4 | 22.4 |
| Total ILCs | 0.043 - 0.66 | 0.19 |
| pDC | 0.16 - 0.77 | 0.49 |
| mDC | 2.73 - 12.4 | 15.2 |
| Total T Cells | 24.8 - 68.5 | 14.2 |
| gd T Cells | 0.78 - 3.68 | 0.16 |
| iNKT | 0.038 - 1.03 | 0.049 |
| CD4^+^ Total Memory | 2.32 - 9.5 | 6.54 |
| CD8^+^ Total Memory | 1.97-4.32 | 0.97 |
