# Supplementary material for: Molecular analysis and immunological characterization of a founder mutation causing ARPC1B deficiency
Source: Genes Immun. 2025 Nov 17;27(1):69–80. doi: 10.1038/s41435-025-00368-w (PMC12923354; doi:10.1038/s41435-025-00368-w)

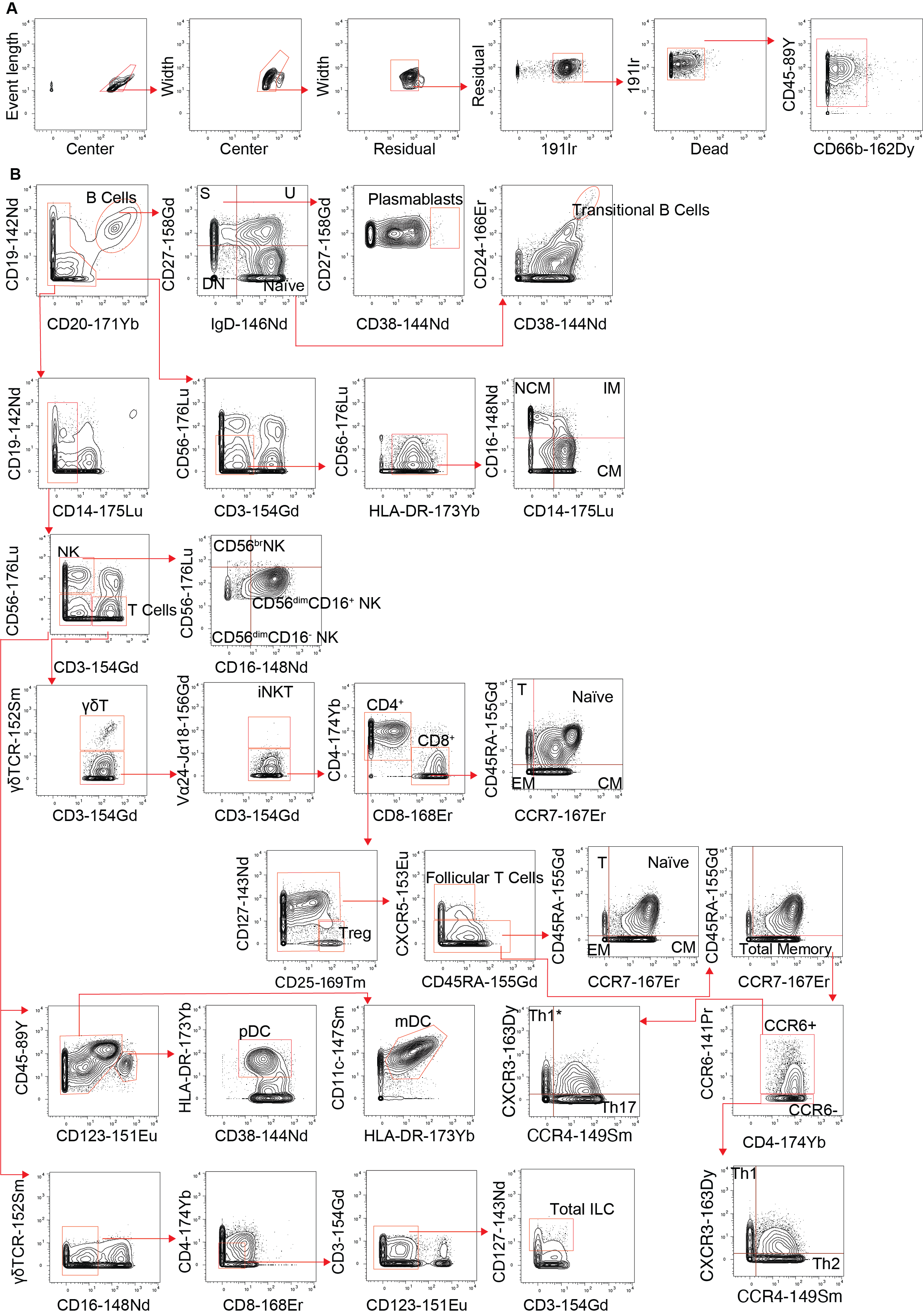
**Supplementary figure 1: CyTOF gating strategy.**

**Supplementary figure 1: CyTOF gating strategy. (A)** Pre-gating strategy before performing CyTOF analysis in R. **(B)** Manual gating strategy continuing from (A) used to identify the different immune cell populations described in this manuscript. In the B cell plots, S correspond to switched, U to unswitched, DN to double negative. In the monocyte plots, NCM to non-classical monocytes, IM to intermediate monocytes, CM to classical monocytes. In the T cell plots EM stands for effector memory, CM for central memory and T for TEMRA.

**Supplementary figure 2: Heat map showing the intensity of markers used to cluster the main populations in Figure 4.**


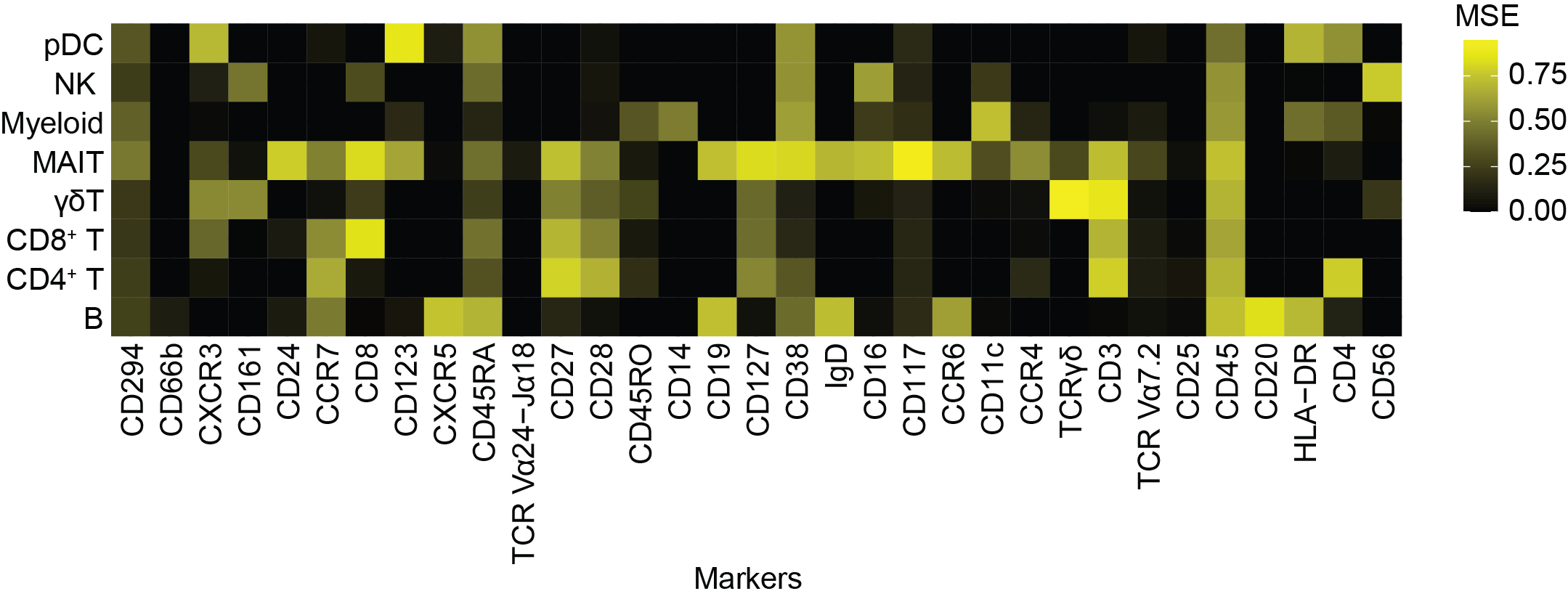

Supplement: Supplementary file 5 — Supplementary material [file 41435_2025_368_MOESM5_ESM.docx]
